# Influence of the Scatter Index of Non-Metallic Inclusions in Structural Steel on the Fatigue Resistance Coefficient

**DOI:** 10.3390/ma16072758

**Published:** 2023-03-30

**Authors:** Tomasz Lipiński

**Affiliations:** Faculty of Technical Sciences, University of Warmia and Mazury in Olsztyn, 10-719 Olsztyn, Poland; tomaszlipinski.tl@gmail.com

**Keywords:** structural steel, inclusions, scatter index, fatigue resistance coefficient, fatigue strength

## Abstract

One of the main parameters characterizing steel is tensile strength. Conducting actual research is time consuming and expensive. For this reason, the technique uses simplified methods that allow one to quickly estimate the resistance of the material to fatigue. They are conducted mainly by computer methods. For the proper development of programs to determine the fatigue parameters of steel, solid data preparation is necessary. Unfortunately, some studies are performed on materials produced in laboratory conditions, which is only an approximation of the actual production conditions. Real alloys contain natural impurities which can affect their properties. Therefore, it is important to use real results obtained on an industrial scale for analysis including computer simulations. One of the important parameters that can be used to describe the properties of steel is the scatter index. It is the quotient of the average distance between the pollution and the average size of the pollution. This parameter makes it possible to take into account the fatigue strength of steel, taking into account the size of impurities and the distance between these impurities. The paper attempted to determine the scatter index and its impact on the fatigue resistance coefficient for steel melted in an industrial 140 ton electric furnace. The tests were carried out on structural steel with an average carbon content of 0.26%. The steel was hardened and tempered in all temperature tempering ranges (low, medium, and high). The fatigue resistance coefficient in the scatter index function was determined and discussed for each of the applied heat treatment parameters.

## 1. Introduction

Non-metallic inclusions play a special role not only during the service of steel, but also during its hardening by means of heat and plastic treatment [1,2,3,4,5]. These treatments may be conducive to the formation of microstructural stresses at the boundary of the precipitates. The influence of impurities on the fatigue strength of metal alloys also depends on local stresses and strains in the microstructure and the mechanisms of nucleation, development, and joining of microcracks. The reasons for the formation of these stresses are differences in the physical properties of the steel microstructures and impurity-forming phases. Other important factors are also the size and distribution of impurities in the volume of steel [6,7,8,9,10,11,12].

In industry, steel heating is carried out in large furnaces. The feed to the furnaces consists of both metal ores and scrap. The occurrence of non-metallic inclusions in steel is completely natural. There are many factors affecting the occurrence of impurities in steel. They can come from, among others, chipping of furnace and ladle components, from contaminated scrap, or they can be formed in the metallurgical process. Elimination of pollutants is economically justified only up to a certain level [13,14,15,16]. For many years, the question of what quantity and quality of impurities are acceptable in metal alloys has been raised. Research in this area gives different answers. The conclusions of these studies show that the acceptable contamination of alloys depends on their intended use [17,18,19,20,21,22,23]. Considering the influence of the same impurities present in low-ductility hard steels [13,24,25,26,27,28,29,30,31,32,33,34,35] and in high-ductility steels [27,28,29,30,31], different effects of impurities on steel properties were noted. Comparing the impact of impurities with a specific quantitative and qualitative structure on the properties of steel given in various scientific works, it can be concluded that in steels with a hard, non-deformable microstructure, the probability of negative impact of impurities is higher [13,32,33,34,35]. Many studies conducted so far on a real scale and on the material obtained in the real production process are replaced by the assessment of their properties based on computer simulations [36,37,38,39,40]. Robust real-world data are essential to develop programs that represent, as closely as possible, the manufacturing processes and properties of metal alloys. It should be emphasized that the results of testing steels produced in industrial conditions have a completely different meaning than those generated by simulations carried out on a small scale in laboratory conditions. It must be admitted that the results of tests on a laboratory scale require appropriate adjustment before being used on an industrial scale.

The literature provides equations and coefficients on the basis of which the fatigue strength of various steel grades can be determined depending on the strength derived from the static tensile test (1) [41].

*z_g_* = *c* · *R_m_*, (1)

There are also well known relationships to determine the static strength of the material based on its hardness (2) [41].

*R_m_* = *d* · *H*, (2)

Based on the abovementioned, it can be assumed that fatigue strength is proportional to hardness and expressed as *k* (3).

(3) $$k=\frac{z_{g}}{H}$$

where:

*z_g_*—fatigue strength, MPa;
*c*—the proportionality coefficient of fatigue strength and tensile strength;
*d*—the proportionality coefficient of tensile strength and hardness;
*R_m_*—tensile strength, MPa;
*H*—hardness, MPa;
*k*—fatigue resistance coefficient.

The subject of the influence of impurities on the parameters describing the fatigue strength of steel is very popular [42,43,44,45,46,47,48,49,50,51,52]. Analyzing the available literature, no analysis of fatigue resistance coefficient was found in the scatter index function, which is the quotient of the average size of contamination and the average distance between contaminations. Few research works also deal with the subject of fatigue strength of high-ductility steel as a function of impurity content. There are also few publications presenting the results of research on an industrial scale, which is important for the development of computer simulation programs [53,54,55]. The abovementioned limitations comprise another confirmation of the need to conduct research in these areas. This attempt was made in this paper.

Based on the analysis of the current state of the issue, it was decided to determine the fatigue resistance coefficient in the scatter index function (average distance between the pollution and the average size of the pollution) for high-ductility structural steel with variable microstructure, and thus different mechanical properties. Estimation of the scatter index made on the basis of tests carried out in industrial conditions and after taking into account the effect of the size of impurities and the distance between impurities allow determination of the fatigue resistance coefficient with greater accuracy.

## 2. Materials and Methods

The tests were carried out on structural steel with the chemical composition shown in Table 1.

The steel was heated under industrial conditions in a 140 ton basic arc furnace. The furnace charge was supplemented with steel scrap. Scrap accounted for about 25% of the total charge weight. The liquid metal was poured into 7 ton ladles and desulfurized. Then, the alloy was rolled into ingots with a square cross-section with a side of 100 mm using classical methods.

Fifty-one sections were taken from the billets to test the fatigue properties. Each of them had the shape of a cylinder, the diameter of which was 10 mm. The direction of the longitudinal axis of the cylinder coincided with the direction of steel rolling. Since the fatigue life depends, among other things, on the hardness and ductility of the steel, it was decided to carry out the tests for different hardnesses and ductilities of the material. For this purpose, before the test, the steel was subjected to heat treatment consisting of hardening with austenitizing at the temperature of 880 °C. Then, the samples were tempered at temperatures of 200 °C, 300 °C, 400 °C, 500 °C, and 600 °C for 120 min. Fatigue strength tests were carried out on a machine enabling rotational bending [56]. A bending frequency of 6000 cycles per minute was used. The stress levels were determined on the basis of preliminary tests, assuming the durability of 10^7^ cycles. The levels of applied stresses depending on the tempering temperature are shown in Table 2.

The chemical composition of steel for each of the heats was determined using a Leco analyzer, ARL FICA quantometer, and conventional methods of analytical chemistry. The total volume of impurities was assessed using the extraction method.

The dimensions of the impurities were estimated at the image analysis station using an inspection video microscope at 400× magnification. Dimensional ranges of impurities larger than 2, 5, and 10 µm were analyzed. The content of impurities with dimensions of up to 2 µm was calculated from the difference between the total volume of impurities and the volume of impurities greater than or equal to 2 µm determined using a video microscope. The analysis was carried out assuming that the quotient of the volume of impurities observed on the elementary surface and the area of this surface is equal to the quotient of the volume of impurities existing in the elementary space and the volume of this space [57]. Since the content of impurities present in the tested steel based on sulfur and phosphorus was lower than the measurement error, their analysis was abandoned.

The scatter index, which is the quotient of the average pollution size and the average distance between the pollution, was determined from Equation (4).

(4) $$\chi =\frac{\lambda }{\overset{-}{d}}$$

where:


(5) $$\overset{-}{\lambda }=\frac{2}{3}\overset{-}{d}\left(\frac{1}{V}-1\right)$$

$\overset{-}{d}$—average diameter of impurities, µm;
*V*—relative volume of impurities, %.

The steel fatigue resistance coefficient *k* for all tempering temperatures used is presented in a general form by Equation (6):

*k*_(*tempering temp*.)_ = *a* · χ + *b*, (6)

where:

*k_(tempering temp.)_*—fatigue resistance coefficient for the assumed tempering temperature;
*a* and *b*—coefficients of the equation;
χ—scatter index.

The significance of the correlation coefficients *r* of each of the regression equations was determined based on the t-Student statistical distribution for the significance level α = 0.05 and the number of degrees of freedom f = n−1.

The dissipation fatigue resistance coefficient *δ* (tempering temperature) for each of the regression equations *k* (tempering temperature) (6) was calculated Equation (7):

(7) $$\delta_{\left(tempering\;temp.\right)}=\frac{2\cdot s}{\sqrt{1-r^{2}}}$$

where:

*s*—standard deviation;
*r*—correlation coefficient.

## 3. Results

**Figure 1:**
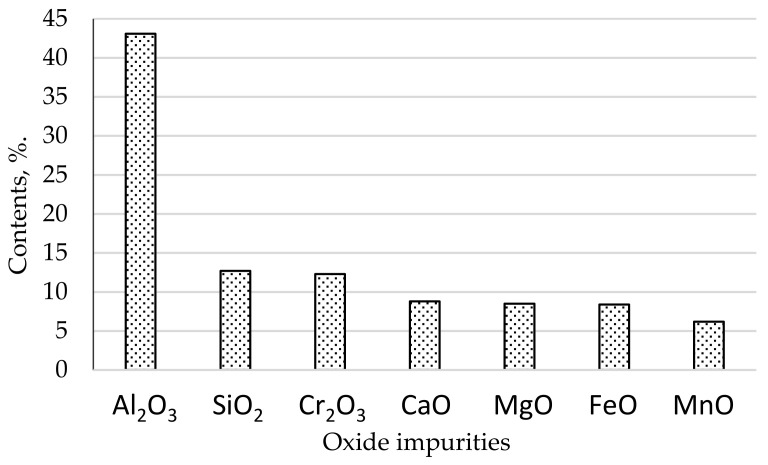
Types of oxide impurities content in the tested steel.

Based on the qualitative metallographic and X-ray spectrum analysis of the metallographic samples performed on a Jeol scanning electron microscope with X-ray analyzer the distribution of impurities for one of the heats was determined (Figure 1).

**Figure 2:**
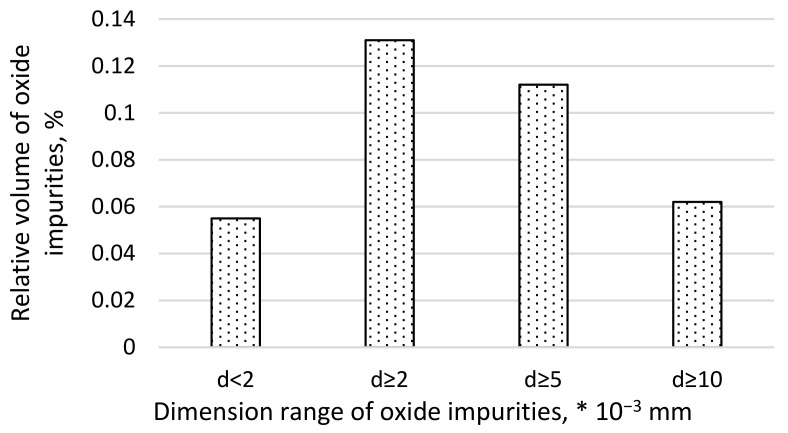
Dimensional structure of steel impurities.

The dimensional structure of impurities for an example heat of the tested steel is shown in Figure 2.

**Figure 3:**
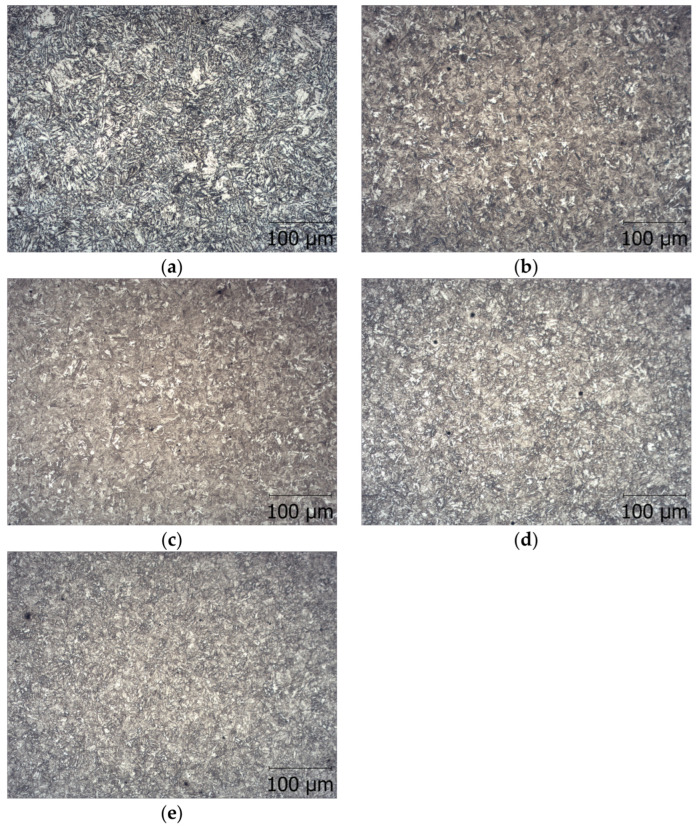
Microstructure of researched steel hardened at 880 °C, and tempered (**a**) at 200 °C, (**b**) at 300 °C, (**c**) at 400 °C, (**d**) at 500 °C, and (**e**) at 600 °C.

The microstructure of hardened steel with austenitizing at 880 °C and then tempering at low, medium, and high tempering temperatures is shown in Figure 3.

Average Vickers hardness of the hardened steel with austenitizing at 880 °C and then tempering at low, medium, and high temperatures is shown in Table 3.

**Figure 4:**
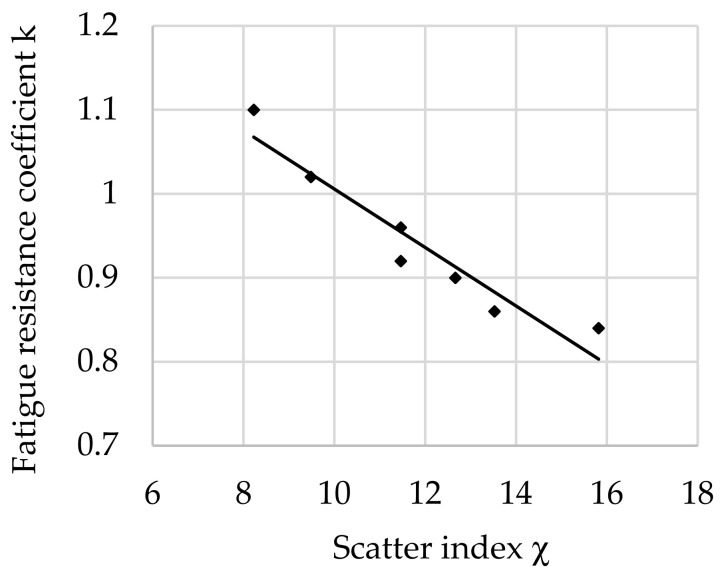
The relationship between the fatigue resistance coefficient and scatter index of structural steel hardened and tempered at 200 °C.

The relationship between the fatigue resistance coefficient and scatter index χ for rotational bending of structural steel hardened at 880 °C and tempered at 200 °C is shown in Figure 4.

The regression equation with correlation coefficient *r* for the tested steel tempered at 200 °C is as follows (8):

*k*_(200)_ = −0.0348 · χ + 1.3541 and *r* = 0.9560 (8)

**Figure 5:**
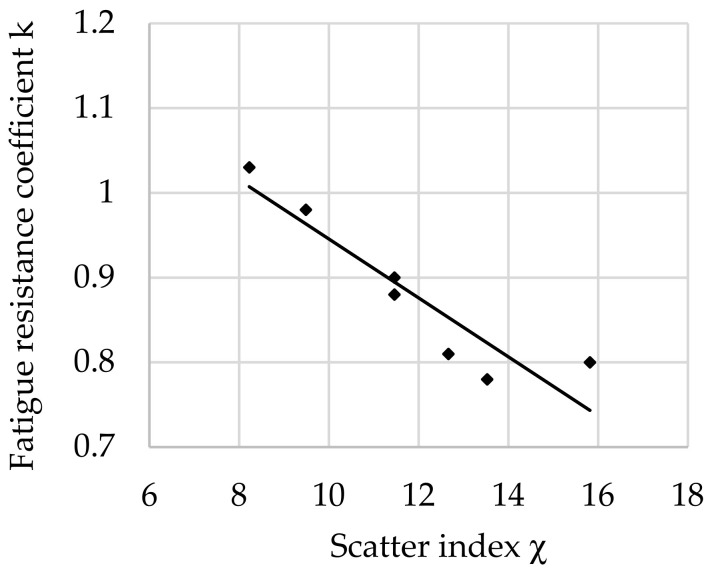
The relationship between the fatigue resistance coefficient and scatter index of structural steel hardened and tempered at 300 °C.

The relationship between the fatigue resistance coefficient and scatter index χ of structural steel hardened at 880 °C and tempered at 300 °C is shown in Figure 5.

The regression equation with correlation coefficient *r* for tested steel tempered at 300 °C is shown as follows (9):

*k*_(300)_ = −0.0348 · χ + 1.2933 and *r* = 0.9237 (9)

**Figure 6:**
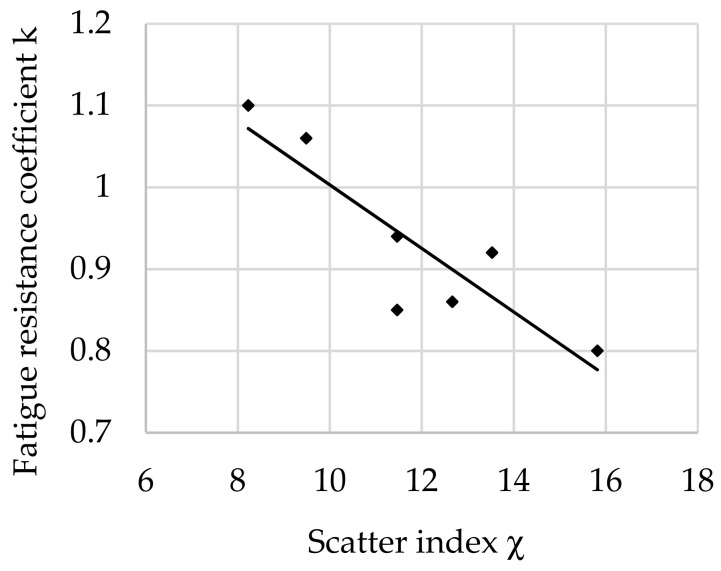
The relationship between the fatigue resistance coefficient and scatter index of structural steel hardened and tempered at 400 °C.

The relationship between the fatigue resistance coefficient and scatter index χ of structural steel hardened at 880 °C and tempered at 400 °C is shown in Figure 6.

The regression equation with correlation coefficient *r* for tested steel tempered at 400 °C is shown as follows (10):

*k*_(400)_ = −0.0388 · χ + 1.3914 and *r* = 0.8819 (10)

**Figure 7:**
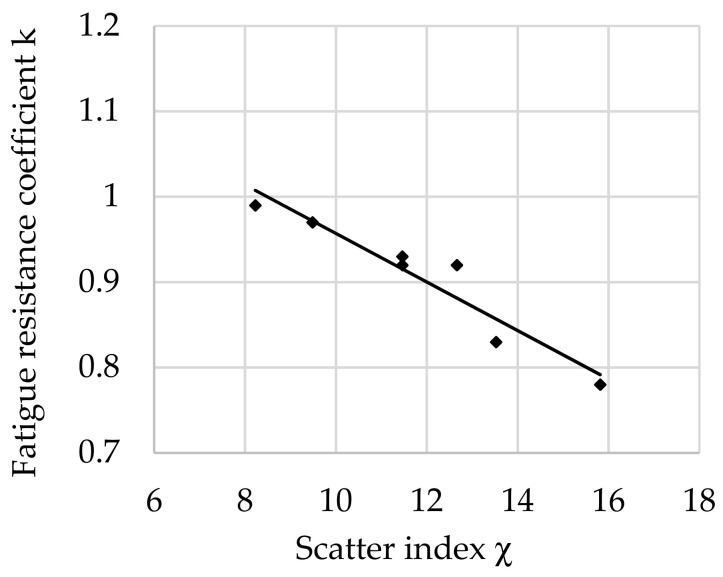
The relationship between the fatigue resistance coefficient and scatter index of structural steel hardened and tempered at 500 °C.

The relationship between the fatigue resistance coefficient and scatter index χ of structural steel hardened at 880 °C and tempered at 500 °C is shown in Figure 7.

The regression equation with correlation coefficient *r* for tested steel tempered at 500 °C is shown as follows (11):

*k*_(500)_ = −0.0284 · χ + 1.2413 and *r* = 0.9564 (11)

**Figure 8:**
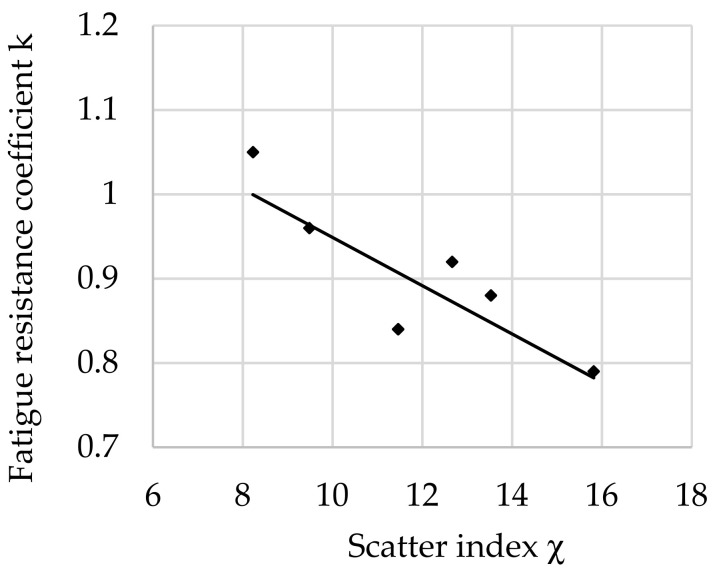
The relationship between the fatigue resistance coefficient and scatter index of structural steel hardened and tempered at 600 °C.

The relationship between the fatigue resistance coefficient and scatter index χ of structural steel hardened at 880 °C and tempered at 600 °C is shown in Figure 8.

The regression equation with correlation coefficient *r* for tested steel tempered at 600 °C is shown as follows (12):

*k*_(600)_ = −0.0286 · χ + 1.2348 and *r* = 0.8232 (12)

**Figure 9:**
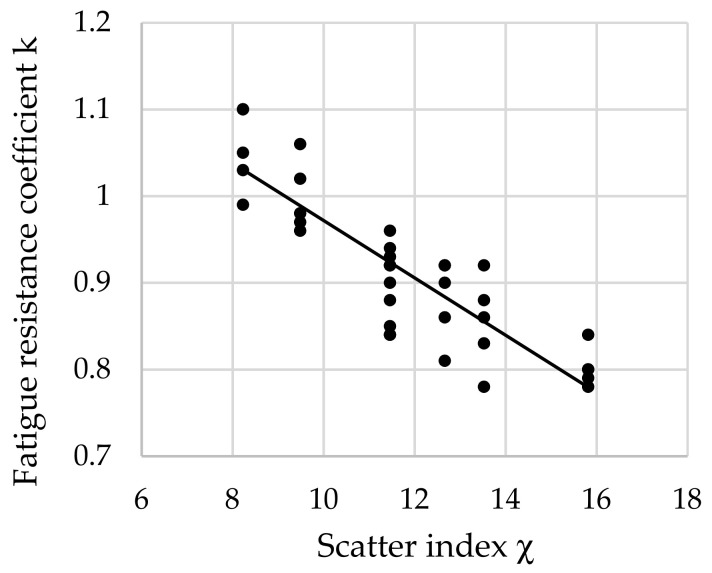
The relationship between the fatigue resistance coefficient and scatter index of structural steel hardened and tempered at all tested temperatures.

The relationship between the fatigue resistance coefficient and scatter index χ of structural steel hardened at 880 °C and tempered at all tested temperatures is shown in Figure 9.

The regression equation with correlation coefficient *r* for tested steel tempered at all tested temperatures is shown as follows (13):

*k*_(all)_ = −0.0331 · χ + 1.303 and *r* = 0.8703 (13)

The statistical parameters for regression Equations (8)–(13) are presented in Table 4.

## 4. Discussion

The tests were carried out on semi-finished products made of high-quality steel. The steel contained an average of 0.26% C and the following alloy additions: manganese, chromium, molybdenum, nickel, silicon, copper, and boron. Sulfur and phosphorus (naturally occurring in steel) were also not avoided, with an average content of 0.02 and 0.011%, respectively. The maximum content of each of them did not exceed 0.023% (Table 1). Despite the attempt to melt steel with the same chemical composition, slight deviations from the assumed values were obtained. This is normal for industrial melts. The standard deviations for the chemical composition are not large and allow the steel melted in all seven melts to be classified as the same grade.

In the analyzed melt, the largest amount of impurities in the microstructure of the tested steel was in the form of Al_2_O_3_ (about 43%). A significantly smaller volume was occupied by non-metallic inclusions in the form of SiO_2_ (about 13%) and Cr_2_O_3_ (about 12%). MeO-type impurities in the form of CaO, MgO, FeO occupied about 8% each, and MnO about 6% (Figure 1).

The relative volume of oxide impurities in the range below 2 µm occupied about 0.06% of the steel volume, larger than 2 µm occupied about 0.13%, larger than 5 µm occupied 0.11%, and impurities considered in the literature as large with a diameter of more than 10 µm occupied 0.06%. Taking into account that large impurities have many times larger volumes than impurities classified to other dimensional ranges, it can be concluded that their amount is small. Analyzing the types of oxide impurities (Figure 1) and their dimension range (Figure 2), it can be indicated with a very high probability that mainly fine impurities with a diameter of up to 2 µm occurred in the steel in the form of Al_2_O_3_. Contaminants with the Al_2_O_3_ and following SiO_2_ and Cr_2_O structure, according to our own observations and those contained in the literature, have a small and compact form [58]. Therefore, they do not constitute sharp structural notches that can significantly reduce the fatigue strength of steel. Therefore, the probability that they will constitute nuclei of discontinuity formation is also low. Based on the data available in the literature, among the impurities found in the tested steel, inclusions containing calcium oxides have the most developed form. However, their share in the steel microstructure is only 8.8%. Taking into account the fact that they should be classified as large precipitates, it can be assumed that they occur in small amounts, so the distance between them is large. Thus, the probability that they will form the nucleus of fatigue microcracks is also low. The abovementioned considerations should, of course, be related to the tested steel, which represents a group of steels characterized by high purity.

By using different variants of tempering, a diverse microstructure of steel was obtained, which is a matrix of non-metallic inclusions with different mechanical properties. The microstructures shown in Figure 3 and the average hardness results shown in Table 3 confirm the different microstructures and their associated hardnesses. The microstructure varied from low-tempered martensite (Figure 3a) with an average hardness of 432 HV through medium-tempered martensite (Figure 3c) with an average hardness of 372 HV to highly tempered martensite (Figure 3e) with an average hardness of 275 HV.

When analyzing the regression equations describing the relationship between fatigue resistance coefficient and scatter index χ, the occurrence of correlation coefficients (8)–(12) were found, confirming the high fit of the equation to the points obtained from the experiment. It should be emphasized that the experiment was carried out on an industrial scale, so such high coefficients testify to the high credibility of the method and research. The lowest correlation coefficient was 0.82 (12) for a tempering temperature 600 °C. The correlation coefficient of the regression equation describing changes in the fatigue resistance coefficient as a dependence on the scatter index, simultaneously for all tempering temperatures, was also high and was set at 0.87 (13).

Analyzing the values of the coefficient a (6) appearing in the regression equations from (8) to (13), a positive directional coefficient was found for all equations describing changes in the *k* coefficient for each tempering temperature. This means directly proportional changes in the coefficient *k* (3) with respect to χ (4). These relationships are visible in Figure 4, Figure 5, Figure 6, Figure 7, Figure 8 and Figure 9.

Coefficient a (6) of Equation (8) for a tempering temperature of 200 °C and Equation (9) for a tempering temperature of 300 °C as well as Equation (11) for a tempering temperature of 500 °C and Equation (12) for a tempering temperature of 600 °C had the same values, so both pairs of functions describing changes in the *k*-factor are parallel. In the range of χ∈< 8; 14> for the tempering temperature of 300 °C, however, a greater angle of inclination of the line describing *k* depending on χ was observed (Figure 5) than for the temperature of 500 °C (Figure 7). This means that a change in χ in this range has a greater effect on the fatigue resistance coefficient. For steel after hardening and tempering at 200 °C, high values of fatigue resistance coefficient *k* were noted depending on the scatter index χ. Therefore, in the heat treatment variant, the dispersion of results is small: correlation coefficient *r* = 0.956 and dissipation fatigue resistance coefficient for *k δ*_(200)_ = 0.05. For the tempering temperature of 600 °C, *r* = 0.9237 and *δ*_(300)_ = 0.0728 were obtained. This confirms reports in the literature of higher reliability of tests for hard steels [5,12,14]. Observing the changes of *k* depending on χ∈<13; 16> after tempering at temperatures of 200 °C and 300 °C (for steel with higher hardness), slight changes in the coefficient *k* were found along with the change in χ. This may mean that in this range, with an increase in the distance between the impurities or a decrease in the diameter of the impurities (i.e., for steel with higher purity), the decrease in the *k* coefficient stops with the increase in χ. This confirms the observations presented in the works of other authors [12,24,45].

After tempering at high temperatures of 500 °C and 600 °C, a smaller decrease in the *k* coefficient with an increase in χ was noted than for steels tempered at low and medium temperatures. Steels tempered at higher temperatures have higher ductility resulting from the microstructure of sorbitol (500 °C) and spheroidite (600 °C), and therefore it is highly probable that the small-diameter non-metallic inclusions present in the microstructure are not the sites of micro-crack initiation. It also seems possible to relieve stresses by inhibiting dislocations on particles and bypassing inclusions by dislocations. This effect certainly reduces local hardening and thus facilitates further deformation, thus increasing the fatigue resistance coefficient *k*.

It should be remembered that there is a relatively large amount of fine impurities in steel (compared with large-sized impurities). Large-volume impurities are very rare in the tested steel. Thus, the results refer to high purity steel containing non-metallic inclusions of small dimensions. An interesting direction for further research seems to be the determination of the impact of very fine impurities on the fatigue resistance coefficient.

## 5. Conclusions

The results of the conducted research are the relationships defining the fatigue resistance coefficient in the scatter index function for high-ductility structural steel with variable microstructure and hardness.

The obtained test results confirmed the strong dependence of the fatigue resistance coefficient *k* on the scatter index χ of structural steel hardened and tempered for all tested tempering temperatures.

With the increase in tempering temperatures, thus obtaining a more ductile microstructure, the influence of impurities (represented by the diameter of the impurities and the distance between them) on the fatigue resistance coefficient decreased.

It was confirmed that the fatigue strength tests of higher hardness steels are subject to a lower error than the tests performed on lower-hardness steels.

It was found that with the increase in the distance between the impurities and/or the decrease in the dimensions of non-metallic inclusions (scatter index χ), the value of the fatigue resistance coefficient *k* increased.

## Figures and Tables

**Table 1:** Average contents of the chemical composition of the tested steel and its standard deviation.

Chemical Element	C wt. %	Mn wt. %	P wt. %	S wt. %	Cr wt. %	Mo wt. %	Ni wt. %	Si wt. %	Cu wt. %	B wt. %
Average contents	0.259	1.183	0.020	0.011	0.524	0.246	0.500	0.236	0.154	0.0027
Standard deviation	0.030	0.190	0.003	0.003	0.030	0.016	0.040	0.069	0.040	0.0008

**Table 2:** Applied load for fatigue strength test for assumed tempering temperatures.

Tempering Temperature	°C	200	300, 400, 500	600
Applied load	MPa	650	600	540

**Table 3:** Average Vickers hardnesses for tested tempering temperatures.

Tempering Temperature	°C	200	300	400	500	600
Vickers hardness	HV	432	412	372	333	275
Standard deviation	HV	21	17	29	23	30

**Table 4:** Dissipation fatigue resistance coefficient, correlation coefficients, and test probability.

Tempering Temperature °C	Dissipation Fatigue Resistance Coefficient *k* (6)	Correlation Coefficient *r*	Test Probability *t_α_* = 0.05	Critical Value from Student’s Distribution for *t_α_* = 0.05 and *p* = (*n −* 1)
200	0.0540	0.956	7.9822	
300	0.0728	0.9237	5.9058	
400	0.1048	0.8819	4.5822	2.4469
500	0.0438	0.9564	8.0213	
600	0.0996	0.8232	3.5516	
all	0.0887	0.8703	10.3035	2.0452

## Data Availability

Not applicable.
